# Learning Representative Features by Deep Attention Network for 3D Point Cloud Registration

**DOI:** 10.3390/s23084123

**Published:** 2023-04-20

**Authors:** Xiaokai Xia, Zhiqiang Fan, Gang Xiao, Fangyue Chen, Yu Liu, Yiheng Hu

**Affiliations:** 1 Beijing Institute of System Engineering, Beijing 100101, China; 2 Artificial Intelligence Institute of China Electronics Technology Group Corporation, Beijing 100041, China; 3 State Key Laboratory of Software Development Environment, Beihang University, Beijing 100191, China; 4 School of Computer Science and Engineering, University of New South Wales, Sydney, NSW 2052, Australia

**Keywords:** point cloud, deep learning, registration, feature extraction

## Abstract

Three-dimensional point cloud registration, which aims to find the transformation that best aligns two point clouds, is a widely studied problem in computer vision with a wide spectrum of applications, such as underground mining. Many learning-based approaches have been developed and have demonstrated their effectiveness for point cloud registration. Particularly, attention-based models have achieved outstanding performance due to the extra contextual information captured by attention mechanisms. To avoid the high computation cost brought by attention mechanisms, an encoder–decoder framework is often employed to hierarchically extract the features where the attention module is only applied in the middle. This leads to the compromised effectiveness of the attention module. To tackle this issue, we propose a novel model with the attention layers embedded in both the encoder and decoder stages. In our model, the self-attentional layers are applied in the encoder to consider the relationship between points inside each point cloud, while the decoder utilizes cross-attentional layers to enrich features with contextual information. Extensive experiments conducted on public datasets prove that our model is able to achieve quality results on a registration task.

## 1. Introduction

**Figure 1:**
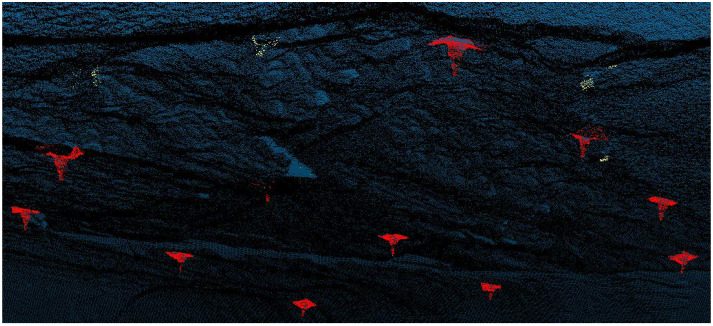
Application of point cloud on bolt detection in the mining industry.

A point cloud is a widely used data structure for 3D object description due to its precision and easy accessibility. Its applications range from autonomous driving to underground mining [1,2,3,4]. Figure 1 depicts a practical example, where points cloud data are adopted in the detection of bolts in the mining industry [1]. Point cloud registration is an important task over point cloud data and aims to locate the optimal transformation that aligns two different point cloud datasets. It is fundamental to many downstream applications, including simultaneous localization and mapping (SLAM), robotics, and 3D reconstruction. For instance, registration can be a useful tool in aligning street views during the construction of smart cities [5]. Due to the various practical applications, the exploration of point cloud registration holds great importance. Traditional point cloud registration methods rely heavily on initialization pose and struggle to handle complex cases with partial or noisy point clouds. Recent advances in deep learning have led to a growing number of learning-based approaches applied to registration tasks.

Existing learning-based techniques for point cloud registration generally employ correspondence-based methods [6,7,8,9,10], where typically three main steps are involved. First, a network is developed to extract features on a pointwise basis. Next, correspondences between two point clouds are identified by assessing feature similarity. Last, a transformation matrix is produced using the acquired correspondences. Of the three steps, the feature extraction phase is especially vital in obtaining high-quality results. In the earlier stage for investigating the point cloud registration problem, handcrafted features such as FPFH [11] and SHOT [12] are commonly utilized to derive pointwise features. On the other hand, more recent studies have focused on deep-learning-based approaches. Most methods aim to learn the spatial encoding of points or the geometric structure of local areas [13,14,15]. While these features have proved useful in identifying the correspondences, they struggle to distinguish points in nonsalient regions, where the point features are not sufficiently discriminative. Consequently, performances are less satisfactory without the availability of shared contextual information across point clouds.

Recently, the attention mechanism has gained popularity as a tool for feature matching. The cross- and self-attention mode, which was proposed by [16], has been shown to be effective in identifying two-view correspondences. This technique ensembles the intuition that when humans attempt to match two images, they frequently compare details by looking back and forth. Similarly, the attention mechanism enables the exchange of information between two items, thereby improving the accuracy of potential matching. The attention approach has been incorporated into numerous computer vision tasks, including registration. However, applying this module directly to raw point clouds can result in computational issues due to the necessity of stacking multiple attentional layers to learn representative features with sufficient contextual information. Unlike 2D images, which have a limited number of pixels, point clouds often contain a large number of points, making it prohibitively expensive to handle the linear computation brought by the attentional layer. In light of this, recent studies attempt to use specific sampling strategies to initially reduce the number of points [17,18]. However, the use of a sampling strategy can significantly impact feature quality since a downsampled point cloud cannot well preserve the original geometric structure. Since most feature extraction models depend on the structure or detail of a local patch to describe a point, point reduction can distort the shape and lose useful information. Additionally, the choice of sampling strategy presents another challenge for the registration task. Therefore, directly downsampling points may not be the optimal solution for addressing the expensive computation costs in attention approaches for the point cloud registration task.

The aforementioned issues have motivated studies to integrate the attention module with a hierarchical feature extraction model to alleviate the high computation costs [10,19,20]. In contrast to prior methods, which compute features directly on the downsampled point cloud, this approach allows each layer to gradually sparsify the points and deepen features based on the previous layer to ensure the preservation of information. However, the efficacy of the attention module may be restricted, as it is usually applied at the last subsampled layer, where only the sparse points at the highest level are utilized in detecting potential matches. This may result in a situation where features propagated to lower levels cannot benefit fully from the advantages of the attention mechanism. Therefore, it is critical to maximize the value of the attention module without incurring expensive computation costs for 3D point clouds.

To address this issue, we propose a model called DAN based on an encoder–decoder framework, which fully capitalizes on the advantages of a multplex graph attention module at a reasonable computational cost and hierarchically derives keypoints and associated features. Instead of solely employing the attention module at the last subsampled points, our method embeds self-attentional layers in the encoder and cross-attentional layers in the decoder. As a result, the encoder is responsible for extracting individual features within the point cloud, while the decoder emphasizes contextual information across point clouds, which can be beneficial in finding potential matches. This approach avoids the problem of contextual information derived from the abstract level not being propagated in the right direction. Furthermore, with the assistance of graph attentional layers, the topological structure of the point cloud can be thoroughly explored, enhancing feature robustness.

## 2. Related Work

In this section, we mainly review the deep learning registration models that adopt correspondence-based methods.

### 2.1. Feature Extraction

Correspondence-based methods usually involve the extraction of pointwise features and then establish correspondences based on feature similarity. Thus, the goal is to find representative features that can identify correspondences from points that share similar characteristics. A revolutionary work, PointNet [13], is proposed to directly process unordered point sets, which is comprised of several shared multilayer perceptron (MLP) layers. It computes the spatial encoding of individual points, which makes it highly efficient. However, it does not take the local structure of point sets into account. To make up such deficiency, PointNet++ [14] is designed to capture the hierarchical structure. The set abstraction operation in PointNet++ provides a systematic way to summarize the local pattern around keypoints by grouping the features of points from the neighborhood.

Inspired by the success of convolutional neural networks (CNNs) in 2D computer vision tasks, many models utilize the idea of CNNs in 3D point cloud feature extraction. Different from rigid formats such as 2D pixels, point clouds are unordered and uneven. Because of these properties, directly adopting the concept of CNNs on 3D point clouds is challenging. One of the solution is to construct a 3D voxel analogous to the grid pattern of 2D pixels. However, the additional dimensionality could induce huge computation cost, which is unfeasible to process large-scale data. Thus, some studies seek alternative ways to define the notion of convolution for 3D point clouds. KPConv [21] is another widely used model for point-cloud-related tasks. It introduces the concept of kernel points, which mimics the functionality of kernel pixels in an image. Ref. [22] devised Edge-Conv, which utilizes graph structure to present point clouds. It gives the flexibility to dynamically capture the topological structure in each layer. Generally, these works can be used in any point-cloud-related task, such as segmentation and classification. However, the discrepancies between registration and other tasks result in different demands on feature extraction.

As the registration task involves the matching of points between two or more point clouds, extracting discriminative features that can help to identify correspondences is usually the main focus. Based on these factors, many works are proposed specifically to address the registration problem. Ref. [15] leveraged a 3D convolutional network to learn the representation of a point from its local region features. The 3D convolutional layer is constructed by the TDF voxel grid around a point to capture the local structure. FCGF [9] constructed a fully convolutional network, where the generalized sparse convolutional layer is defined by [23] along with sparse tensors. It is capable of processing high-dimensional data effectively, since it can reuse intermediate activations on overlapping areas. Ref. [6] proposes a method to utilize joint learning on point clouds. The model produces a descriptor and a detection score for each point. The detection score is capable of finding the keypoints that are more discriminative, which makes it easier to identify correspondences. In contrast with previous methods which are more generalized, these methods often produces more distinctive features that can aid in the search for correspondences. They often use contrastive loss to train pointwise features, as it can guide the model to minimize the similarity between correspondences and maximize the similarity between points that cannot be matched together.

### 2.2. Attention Mechanism

Inspired by the success of transformers in NLP, many studies began to adopt attention mechanisms in computer vision tasks, including point cloud registration. The usage of attention mechanisms can help to enrich the features from a different perspective. As the first work that embeds a transformer module into the model, DCP [24] provided the functionality of contextual aggregation that considers the relations between two input point clouds, instead of embedding them independently. Another model, ref. [25], proposes a method that utilizes the transformer in a different way. It firstly constructs a graph from the point cloud by seeing points as nodes. Then, it employs the transformer into the edge generator to explore the relationship between points with the aid of contextual information.

To thoroughly utilize the advantage of attention mechanisms in computer vision tasks, SuperGlue [16] developed a multiplex graph neural network to solve the two-view correspondences problem, where the keypoints are defined as nodes and the connections are defined as edges. The message passing is carried out via alternating cross- and self-attention. As SuperGlue has achieved remarkable results, some studies attempt to adapt this module onto point cloud registration tasks. Since directly using attention mechanisms on raw point clouds tends to be infeasible for large-scale datasets due to the computation cost, refs. [17,18] seeks different sampling strategies to mitigate this issue. Fischer, K. et al. [17] introduced a pillar layer, which encodes a set of dense points by selecting a sparse subset of keypoints. Shi, C. et al. [18] make use of an existing pretrained model [26] to detect keypoints first.

Another form of using attention modules without a reduction in points was firstly proposed by [10]. It devises a method that embeds the attention module between encoder and decoder. It provides the functionality of detecting overlapping points and their matchability. Following this work, ref. [20] proposed a model that predicts rigid transformation via a closed-form solution where the correspondence loss is able to be taken into account, as it provides a differentiable formula to estimate the distance between the transformed point and the target point. These methods use an attention module on superpoints which has been downsampled and then propagate to original dense points. However, the computed features of superpoints might not be able to contain enough geometric information. To this end, ref. [19] aims to devise a model that improves the accuracy of matchings between superpoints by considering the geometric structure, rather than only making use of abstract embeddings.

Attention modules have been proved to be a useful tool in registration tasks, as they have the capability of obtaining contextual information which other methods are not able to provide. However, for the aforementioned methods, the result relies on the superpoint operations, where the power of the attention mechanism might be diminished during propagation.

## 3. Methods

Given two point clouds $P=\{p_{i}\in R^{3}|i=1,\cdots ,N\}$ and $Q=\{q_{j}\in R^{3}|j=1,\cdots ,M\}$, the registration task aims to find the optimal transformation *T* that could align point cloud *P* and *Q*. *T* can be represented by

(1) $$T=\left[\begin{array}{cc} R & t\\ 0 & 1 \end{array}\right],$$

where R∈SO(3) and $t\in R^{3}$. For correspondence-based methods, the goal is to solve the following:

(2) $$\min_{R,t}\sum_{(p_{i},q_{j})\in C}||Rp_{i}+t-q_{j}\left|\right|,$$

where C is a set of true correspondences. Our model aims at identifying the correspondences based on the similarity of feature points.

Inspired by [21], our model adopts an encoder–decoder framework which extracts points and associated features in a hierarchical manner. In this section, we first describe the basic operations within the encoder and decoder in our model. Next, we briefly explain the attentional layers and how they are embedded in the model.

### 3.1. Feature Extraction

**Figure 2:**
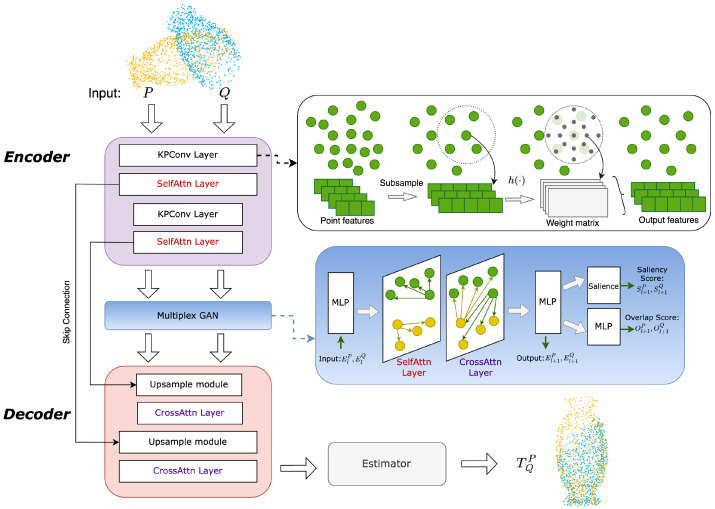
Architecture of the proposed model.

As shown in Figure 2, the encoder is established by alternating KPConv layer and the self-attention layer. Through this approach, we can retrieve the basic multilevel features. The embeddings of point cloud *P* at the $l^{th}$ level, denoted by $E_{l}^{p}$, is derived by

(3) $$\begin{array}{cc} E_{l}^{\prime p} & =Conv(E_{l-1}^{p},P_{l-1}), \end{array}$$

(4) $$\begin{array}{cc} E_{l}^{p} & =SelfAttn(E_{l}^{\prime p}). \end{array}$$

where $E_{l}^{\prime p}$ is the intermediate results of the embeddings; $P_{l-1}$ is the subsampled point cloud at level l−1; Conv(·) and SelfAttn(·) represent the KPConv layer and self-attention layer, respectively.

For the KPConv layer, following [21], the convolution operation is defined on a set of kernel points. We denote a point as *x*. The point convolution F with the kernel *g* on the point *x* can be presented by

(5) $$\left(F*g\right)\left(x\right)=\sum_{x_{i}\in N}g\left(x_{i}-x\right)f_{i},$$

where $N=\{||x_{i}-x||\leq r\}$ is a set of neighbors of *x* that lies in the radius *r*. Say we introduce a set of kernel points $\left\{{\tilde{x}}_{k}|k<K\right\}$ and their weight parameter $W_{k}$ that maps input dimension to output dimension. For $y_{i}=x_{i}-x$, the kernel function can be further defined as

(6) $$g\left(y_{i}\right)=\sum_{k<K}h\left(y_{i},{\tilde{x}}_{k}\right)W_{k}.$$

h(·) is the function that describes the correlation between $y_{i}$ and ${\tilde{x}}_{k}$ as following:

(7) $$h\left(y_{i},{\tilde{x}}_{k}\right)=\max\left(0,1-\frac{\left|\right|y_{i}-{\tilde{x}}_{k}\left|\right|}{\sigma }\right).$$

In the decoder stage, the model aims to enrich the feature by contextual information. At each level, it adopts the cross-attentional layer between two point clouds and then propagates the features back to the lower level. During the propagation, we apply skip connection from embeddings in the encoder stage at the corresponding level. We denote the features of point cloud *P* at level *l* as $F_{l}^{P}$. The computation of features is as following:

(8) $$\begin{array}{c} F_{l}^{\prime P},F_{l}^{\prime Q}=CrossAttn\left(F_{l+1}^{P},F_{l+1}^{Q}\right), \end{array}$$

(9) $$\begin{array}{c} F_{l}^{P}=Upsample\left(F_{l}^{\prime P},E_{l}^{P},P_{l}\right), \end{array}$$

(10) $$\begin{array}{c} F_{l}^{Q}=Upsample\left(F_{l}^{\prime Q},E_{l}^{Q},Q_{l}\right). \end{array}$$

where $F_{l}^{\prime P},F_{l}^{\prime Q}$ are the intermediate results; CrossAttn(·) and Upsample(·) are the cross-attention layer and propagation layer, respectively.

### 3.2. Attentional Layer

The main process of multiplex graph attention network is shown in Figure 2. Firstly, the input would be processed by an MLP layer which maps the features into graph embeddings. We denote the embeddigs of point cloud *P* and *Q* at layer *l* as $F_{l}^{p}$ and $F_{l}^{q}$. In the network, the embeddings are updated as following:

(11) $$F_{l+1}=F_{l}+MLP\left(F_{l},m_{l}\right).$$

Next, we introduce the derivation of message $m_{k}$ by attentional aggregation. For simplicity, we omit the label for layers in the following. Firstly, the query, key, and value are retrieved via linear projection:

(12) $$\begin{array}{c} q=W^{q}X+b^{q}, \end{array}$$

(13) $$\left[\begin{array}{c} k\\ v \end{array}\right]=\left[\begin{array}{c} W^{k}\\ W^{v} \end{array}\right]Y+\left[\begin{array}{c} b^{k}\\ b^{v} \end{array}\right],$$

where *W* and *b* are the parameter matrix and bias term at $l^{th}$ layer; *X* and *Y* denote the query point cloud and the source point cloud. To support the multiplex graph attentional module, two modes are available here. The first is self-attention, where *X* and *Y* represent the embedding of the same point cloud. This allows the model to find the internal correlation between points. The other mode is cross-attention, where *X* and *Y* are different point clouds. Through the cross-attention mode, it is capable of detecting the relationship among the points across two point clouds and possibly searching for potential matchings. After the computation of query, key, and value, the scaled dot-product attention value between point *i* and *j* can be computed by

(14) $$a_{ij}=softmax\left(\frac{q_{i}k_{j}^{T}}{\sqrt{d_{k}}}\right),$$

where $d_{k}$ is the dimension of queries and keys. Finally, the message of a point *i* can be derived:

(15) $$m_{i}=\sum_{j}a_{ij}v_{j}.$$

In addition to the intermediate attention module, we apply the self-attention layer in the encoder and the cross-attention layer in the decoder.

Following [10], two extra dimensions are computed by projection on derived features. The first one is the overlap score *O*, which is to predict whether a point lies in the overlapping region. The other one, saliency score *S*, evaluates the probability of finding a matching point. They are computed by the following:

(16) $$\begin{array}{cc} O & =MLP(F) \end{array}$$

(17) $$\begin{array}{cc} S & =softmax(\frac{\langle F^{P},F^{Q}\rangle }{t}), \end{array}$$

where *t* is the weight to scale the effect of softmax.

### 3.3. Loss Function

In this study, we adopt feature loss, overlap loss, and saliency loss to supervise the learning of the model.

#### 3.3.1. Feature Loss

Our model utilizes similarity optimization to learn distinctive features by minimizing the similarity between points that are close to each other and maximizing the similarity between points that are far away from each other. Circle loss [27] is adopted here:

(18) $$L_{feat}=\log\left[1+\sum_{j=1}^{L}e^{\gamma \alpha_{n}^{j}\left(s_{n}^{j}-\Delta_{n}\right)}\cdot \sum_{i=1}^{k}e^{-\gamma \alpha_{p}^{i}\left(s_{p}^{i}-\Delta_{p}\right)}\right]$$

where $s_{p}$ is the similarity between features of close points and $s_{n}$ is for nonclose points; $\Delta_{n}$ and $\Delta_{p}$ are the margins for correspondences and noncorrespondences; γ is the scale factor; and weights are defined as $\alpha_{n}^{j}=s_{n}^{j}-\Delta_{n}$ and $\alpha_{p}^{i}=s_{p}^{i}-\Delta_{p}$. In contrast with other similarity losses, circle loss can be more flexible, as it gives different penalty strengths for between-class similarity and within-class similarity.

#### 3.3.2. Overlap Loss

Our model also computes the overlap loss to learn the probability of a point lying in the overlapping area. The overlap loss function is defined as

(19) $$L_{overlap}=\frac{1}{N}\sum_{i=1}^{N}o_{i}\log{\hat{o}}_{i}+\left(1-o_{i}\right)\log\left(1-{\hat{o}}_{i}\right)$$

where *i* is the index of the correspondence; $o_{i}$ is the ground truth label that is computed by

(20) $$\begin{array}{c} o_{i}=\left\{\begin{array}{cc} 1 & \mathrm{if}\;||T_{P}^{Q}\left(p_{i}\right)-q_{j}{\left|\right|}_{2}\leq r_{o},\\ 0 & \mathrm{otherwise} \end{array}\right. \end{array}$$

where $q_{j}$ is the nearest point of transformed point $p_{i}$ in point cloud *Q*; *r* is the threshold to determine the maximal distance between matching points.

#### 3.3.3. Matchability Loss

To identify whether a point is able to find its correspondences from learned features, the matchaility loss should also be taken into consideration.

(21) $$L_{matchability}=\frac{1}{N}\sum_{i=1}^{N}s_{i}\log{\hat{s}}_{i}+\left(1-s_{i}\right)\log\left(1-{\hat{s}}_{i}\right)$$

The ground truth label $s_{i}$ is defined as

(22) $$\begin{array}{c} o_{i}=\left\{\begin{array}{cc} 1 & \mathrm{if}\;||T_{P}^{Q}\left(p_{i}\right)-q_{j}{\left|\right|}_{2}\leq r_{m},\\ 0 & \mathrm{otherwise} \end{array}\right. \end{array}$$

where $q_{j}$ is a point in *Q* that has the closest features with $p_{i}$.

## 4. Experiments

In this section, we display and analyze the results of different experiments. We select ICP [28], FGR [29], DeepGMR [30], and Predator [10] for comparison on partial-to-partial and noisy point clouds. The first two methods are traditional mainstream registration methods, where we adopt the implementation provided by Open3D [31]. DeepGMR is a registration method that leverages probability distributions over points to achieve robustness against noise. As the proposed method relies on the establishment of correspondences, we chose to include deepGMR as a baseline model to provide a comparison with a correspondence-free method. Additonally, we include Predator, which is one of the most outstanding attention-based models. The key difference between the proposed model and Predator lies in the incorporation of attentional networks. Specifically, Predator employs an intermediate attention module at the highest level of sampled points and features, while our method embeds attentional layers within both the encoder and decoder. In the experiments, we adhere to the code and the pretrained models they provided on ModelNet40. As the training categories were not specified in deepGMR and Predator, we utilized the results from another graph-attention-based network, GAN [32] to make comparisons on unseen categories as it employed the sample settings and protocols.

To assess the transformation result, we use the mean abolute error (MAE) and root mean squared error (RMSE) for rotation (°) and translation. The formulas for MAE and RMSE are as follows:

(23) $$\begin{array}{cc} \mathrm{MAE} & =\frac{1}{N}\sum_{i=1}^{N}\left|y_{i}-{\hat{y}}_{i}\right|, \end{array}$$

(24) $$\begin{array}{cc} \mathrm{RMSE} & =\sqrt{\frac{1}{N}\sum_{i=1}^{N}{\left(y_{i}-{\hat{y}}_{i}\right)}^{2}}, \end{array}$$

where *y* is the ground truth value and $\hat{y}$ is the predicted value. Better performance is indicated by lower values of MAE and RMSE. For each experiment, we take the average of MAE or RMSE over all point clouds in the test dataset as the result.

In the figures displaying qualitative outcomes, the source and target point clouds are represented by yellow and blue points, respectively.

### 4.1. Dataset

In this study, we conduct experiments on ModelNet40 [33], which is a benchmark dataset that includes the CAD models of 12,311 objects from 40 categories. For the experiments, we firstly downsample each point cloud to 1024 points. The target point cloud is obtained by applying transformation to the source point cloud. For the transformation, rotation and translation perturbation is set to 45° and 0.5, respectively.

### 4.2. Comparison on Different Estimators

Firstly, to examine which estimator yields the best performance in the registration task, we conduct experiments on the following different estimation methods:

- **prob**: The interest points are sampled by probabilistic sampling, where the probability is generated by the product of overlap score and saliency score. Subsequently, RANSAC is utilized to perform feature marching and obtain the transformation matrix.
- **topk**: Firstly, a similarity matrix is derived based on the Euclidean distance between the features of points from two point clouds. The top *k* pairs of points with the highest similarity are selected. Next, similar to *prob*, we conduct feature matching on those selected pairs.
- **topk-corres**: It is similar to the *topk* approach. However, instead of conducting feature matching on those points, the transformation matrix is directly computed by applying RANSAC on the top *k* correspondences found in the similarity matrix.
- **topk-kabsch**: It is similar to *topk*. The difference is that the correspondences and their similarity scores are used as the input to the kabsch algorithm, which provides a closed-form solution for computing the transformation matrix.

We evaluate the performance of different estimators with a varying number of points. The mean absolute errors are displayed in Table 1. The experimental results reveal that the best performance for most of the estimators is achieved when the number of keypoints is around 400. Compared with other estimators, the performance of probabilistic sampling is more sensitive to the number of keypoints, especially around 500 to 600. Unlike other methods that mainly rely on similarity, probabilistic sampling relates to the accuracy of the saliency score. The outcomes suggest that when more keypoints are involved, it can be increasingly difficult to identify accurate correspondences among the sampled points given the probability that is relevant to the overlap score and saliency score. In contrast, the increase in keypoints does not have a significant impact on topk-related methods. Notably, the *topk* estimator with RANSAC based on correspondences outperforms all other estimators. It suggests that utilizing the correspondences generated by our proposed method directly could potentially result in higher robustness.

### 4.3. Performance on Partial-to-Partial Point Cloud

**Figure 3:**
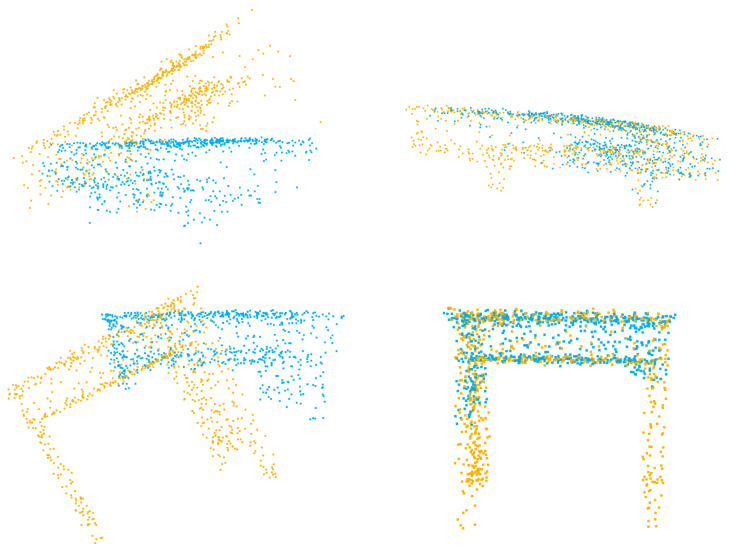
Qualitative results of partial-to-partial point clouds.

The experiment on partial-to-partial point clouds are crucial, as it represents one of the most common scenarios in registration tasks. Table 2 presents the experimental results, where in the experiment settings, 70% of the points are kept for both the source and target point cloud. It is evident that ICP, FGR, and deepGMR struggle to produce quality results when processing cropped point clouds. In contrast, Predator and our method exhibit better performance, as the registration errors shown in Table 2 are much smaller. It is due to the utilization of overlapping scores, which enables both methods to identify and particularly align overlapping regions. Here, our method yields lower registration errors compared with Predator, indicating its better performance in handling cropped point clouds. The qualitative results are demonstrated in Figure 3. Even if partial point clouds are presented, our method is still capable of achieving accurate alignment between two point clouds.

### 4.4. Performance on Point Clouds with Gaussian Noise

**Figure 4:**
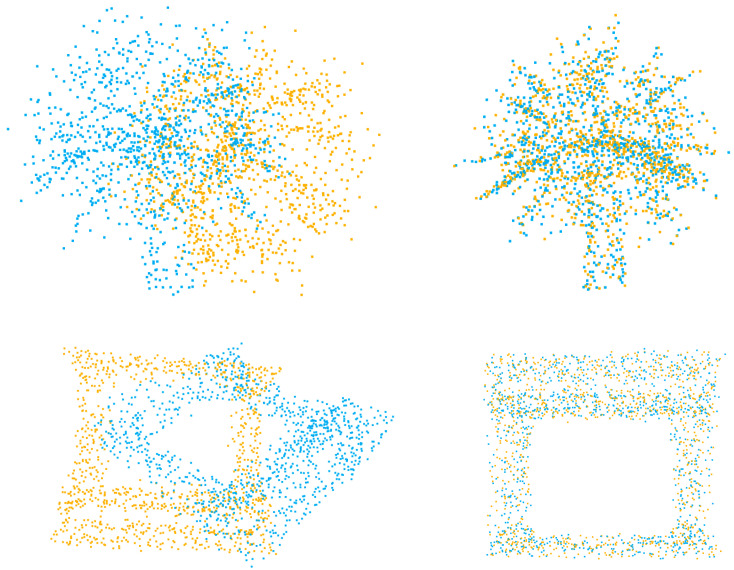
Qualitative results of point clouds with Gaussian noise.

The results of point clouds with Gaussian noise are shown in Table 3, where the noise is sampled from Gaussian distribution, where the mean is 0 and the standard deviation is 0.01. In contrast to the results on partial-to-partial point clouds, all models except ICP achieve better performance in this scenario. This finding suggests that the presented methods are more effective in processing complete point clouds and are relatively robust to noise. Our method shows the highest performance across all evaluation metrics. Figure 4 shows the qualitative results. Despite the addition of noise increasing the difficulty of finding accurate correspondences, our method is still able to achieve high-quality registration results.

### 4.5. Statistical Significance Test

The results in Section 4.3 and Section 4.4 demonstrate that Predator and our model achieved the most satisfying results. Therefore, we conducted a statistical significance test on the registration errors of point clouds in the testing set from Predator and our model. Table 4 reports the *p*-value of different metrics. Here, SE and AE represent squared error and absolute error, respectively, for rotation and translation. For all metrics, *p*-value is smaller than 1%. This indicates that there exists statistical difference between the results derived from our model and Predator.

### 4.6. Performance on Point Clouds from Unseen Categories

**Figure 5:**
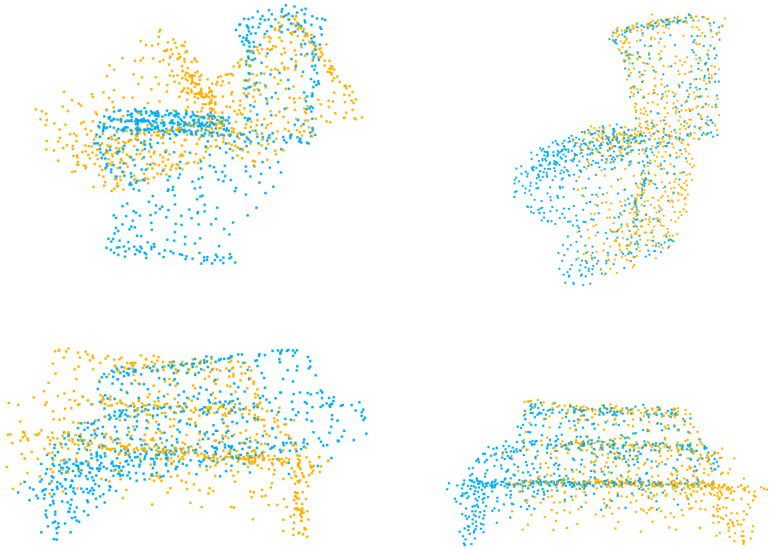
Qualitative results of point clouds from unseen categories.

We conduct experiments on unseen categories, where the categories of point clouds in the test set differ from those in the training set. For learning-based models, the first 20 categories are used for training, and the test is for testing. The result is presented in Table 5, which demonstrates that our model can achieve good performance for all evaluation metrics, similar to the performance achieved in other experiments. It is noteworthy that our model exhibits low variance across difference experiments. Specifically, the variation of rotation MAE is less than $1^{\circ }$, and the translation MAE is less than 0.01, which indicates the ability of our model to handle different registration scenarios. The qualitative result is demonstrated in Figure 5. Even if the categories of source and target point clouds do not appear in the training dataset, our method is still able to accurately align them.

### 4.7. Additional Experiments on Additive Noise

**Figure 6:**
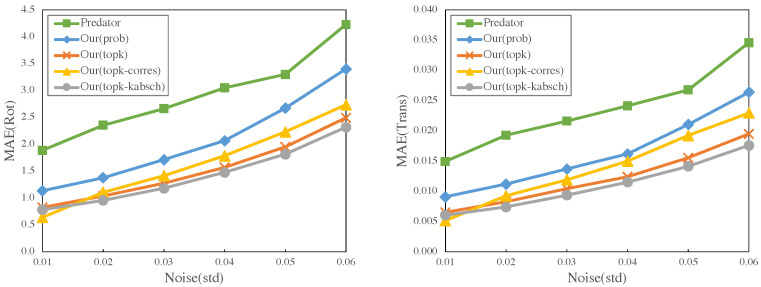
Results on additive noise and cropped data. It displays the MAE of rotation (°) and translation versus the standard deviation of noise.

In this section, to evaluate the robustness of our model, we conduct further experiments with additive noise. Given that ICP, FGR, and deepGMR yielded larger errors, we limited the investigation to Predator and our model with different estimators for better comparison. Figure 6 demonstrates the MAE of rotation and translation for different standard deviations of noise. All of our methods achieve better results than Predator under different amounts of noise. We noticed that most of the estimators on our model have similar performances, where the MAE of rotation is less than 3° and translation is smaller than 0.03. Another observation is that although in Section 4.2 *topk-corres* achieves the best results, its performance is slightly worse than *topk-kabsch* when the amount of noise increases.

### 4.8. Runtime Analysis

As Predator and our method showed superior performance among all the baseline models, we conducted a runtime analysis to compare their efficiency. The test dataset was run under the same conditions, and the overall runtime was divided by the size of the dataset to calculate the runtime of each model. The result is displayed in Table 6. Most of our methods are slightly higher than the computation time of Predator, while the one with *topk-kabsch* as the estimator had lower runtime than Predator. The essential reason is that the kabsch algorithm provides a closed-form solution, while RANSAC requires an iterative process to sample and estimate parameters. As the results shown in Figure 6 indicate *topk-kabsch* achieved the lowest MAE most of the time, we argue that our model is able to produce quality and efficient results with the kabsch algorithm as the estimator.

## 5. Conclusions

In this paper, we propose a model to address the point cloud registration problem. To optimize the advantage of the attention mechanism while keeping computation costs reasonable, our model adopts an encoder–decoder framework with an embedded attentional layer in both the encoder and decoder. The encoder employs self-attention to capture the internal topological structure of individual point clouds, while the decoder uses cross-attention to enhance features by leveraging co-contextual information across point clouds, thereby emphasizing potential match detection. From the experiment results, our model achieved great performance on ModelNet40. However, there is still room for further improvement when it comes to handling registration tasks with other data or scenarios. At the current stage, we only focused on a synthetic dataset of objects to validate the effectiveness of our model. To examine the ability of our method in real cases, we intend to expand the scope to large-scale data, such as the real data of indoor or outdoor scenes captured by laser scanning. Given that KPConv [21] has been validated in datasets with real scenes, it is reasonable to expect that our method, which employs KPConv as its backbone model, will have the potential to handle real data as well. Moreover, as our method utilizes multiple attention layers, its applicability to large-scale data can be challenging due to the significant linear computation involved. Currently, real-time registration is critical for many downstream tasks, such as robotics and autonomous driving. However, achieving real-time performance with high-quality results can be challenging. In the future, we aim to investigate methods for reducing the computational overhead of our approach or allowing parallel computation on large-scale data while retaining its performance.

## Figures and Tables

**Table 1:** Mean absolute error of rotation (°) and translation from different estimators with various number of keypoints.

#Keypoints	100	200	300	400	500	600
prob	2.7544/0.05226	2.3391/0.04305	2.0229/0.04081	2.3391/0.01939	4.2302/0.03271	10.5938/0.07608
topk	1.6285/0.03398	1.5996/0.03365	1.6234/0.03499	1.6099/0.03423	1.6323/0.03537	1.6394/0.03498
topk-corres	1.3452/0.03736	1.2001/0.03337	1.1640/0.03298	1.0684/0.03286	1.0878/0.03409	1.0931/0.03395
topk-kabsch	1.6367/0.03298	1.6119/0.03329	1.6111/0.03340	1.6048/0.03340	1.6085/0.03356	1.6179/0.03357

**Table 2:** The results of partial-to-partial point clouds.

Method	RMSE (R)	RMSE (t)	MAE (R)	MAE (t)
ICP	29.0295	0.9938	22.6922	0.8716
FGR	58.3071	0.3122	36.8645	0.2096
DeepGMR	87.6945	0.4001	66.0553	0.2933
Predator	9.1573	0.0687	3.4375	0.0290
Our	2.8160	0.0318	1.1054	0.0102

**Table 3:** The results of point clouds with Gaussian noise.

Method	RMSE (R)	RMSE (t)	MAE (R)	MAE (t)
ICP	29.7609	0.9931	23.0322	0.8704
FGR	40.3478	0.1910	19.3586	0.1025
DeepGMR	45.8762	0.1877	23.5213	0.09801
Predator	4.6180	0.0371	1.8349	0.0144
Our	1.8833	0.0138	0.6241	0.0050

**Table 4:** The *p*-value of squared error and absolute error for rotation and translation. The first row presents the results for partial-to-partial registration, while the second row presents the registration results for point clouds with Gaussian noise.

Experiment	SE (R)	SE (t)	AE (R)	AE (t)
Partial	$1.24\times 10^{-9}$	$2.25\times 10^{-8}$	$4.32\times 10^{-30}$	$8.74\times 10^{-33}$
Noisy	$9.44\times 10^{-7}$	$4.87\times 10^{-8}$	$4.07\times 10^{-24}$	$6.14\times 10^{-30}$

**Table 5:** The results of point clouds with unseen categories.

Method	RMSE (R)	RMSE (t)	MAE (R)	MAE (t)
ICP	29.0295	0.9938	22.6922	0.8716
FGR	58.3623	0.3034	37.1349	0.2086
GAN	5.977	0.046	4.534	0.021
Our	3.1371	0.0314	1.2608	0.0117

**Table 6:** The runtime of our model with different estimators and Predator.

Method	Our (Prob)	Our (Topk)	Our (Topk-Kabsch)	Our (Topk-Corres)	Predator
Time (ms)	49	50	41	55	43

## Data Availability

The data is openly available in [33].
